# Overproduction of Clavulanic Acid by UV Mutagenesis of *Streptomyces clavuligerus*

**Published:** 2010

**Authors:** Hassan Korbekandi, Parisa Darkhal, Zohreh Hojati, Daryoush Abedi, Javad Hamedi, Meraj Pourhosein

**Affiliations:** a *Genetics and Molecular Biology Department, Isfahan University of Medical Sciences, Isfahan, Iran.*; b *Pharmaceutical Biotechnology Department, Isfahan University of Medical Sciences, Isfahan, Iran.*; c *Genetics Department, Isfahan University, Isfahan, Iran.*; d *Biology Department, Tehran University, Tehran, Iran.*

**Keywords:** *Streptomyces clavuligerus*, Clavulanic acid, UV mutagenesis, Overproduction, Fermentation, UV irradiation

## Abstract

Clavulanic acid is produced industrially by fermentation of *Streptomyces clavuligerus *and researches have increased its production by strain improvement, recombinant DNA technology, and media composition and growth condition optimization. The main objective of this study was to increase the level of clavulanic acid production from *Streptomyces clavuligerus *(DSM 738), using UV irradiation. After incubation, the spores and aerial mycelia were scraped off the agar plate by a sterile loop. After passing through a cotton wool, the serially diluted spore suspension was spread on GYM- agar containing caffeine. The plates were irradiated with UV light, wrapped in aluminum foil and incubated. The colonies were sub-cultured again to express the mutations. An aliquot of the spore suspension prepared from the resulted culture was poured in GYM agar plates and incubated. The plates were overlaid with nutrient-agar containing penicillin G and *Klebsiela pneumoniae*, and incubated. The inhibition zone diameter was measured and compared with the wild type colony. Repeating this procedure, the overproducer mutants were selected. Concentration of clavulanic acid was determined by HPLC analysis. It was concluded that secondary metabolites, mainly antibiotics containing clavulanic acid, were produced about 6–7 days after the growth, and concentration of clavulanic acid was increased up to two-folds after UV mutagenesis.

## Introduction

*Streptomyces clavuligerus *is an actinomycet, which produces more than 21 bioactive secondary metabolites (1-3). Between them, metabolites with β-lactam ring such as cephamycyn C, penicillin N and cephalosponins with antibacterial effect, and clavulanic acid, a β-lactamase inhibitor, are more important (4). Clavulanic acid is a β-lactam antibiotic with weak antibacterial effect and strong β-lactamase inhibition (4-6). Augmentin^®^, a brand name, containing a combination of amoxicillin and potassium clavulanate, is one of the best selling antibiotics (7-15). Clavulanic acid is produced industrially by fermentation of *Streptomyces clavuligerus *and researches have increased its concentration (up to ≈1.4 g L^-1^) by strain improvement, recombinant DNA technology, and media composition and growth conditions optimization (3, 7, 10, 15-24). UV irradiation is one of the strain improvement strategies through random mutation (7, 16). UV radiation, in the range of 200-300 nm, produces thymidine dimmers and increases probability of deletion during the duplication process (25, 26). UV is a very convenient and relatively safe mutagen (27), however very few researchers (7, 16) have used this technique to overproduce clavulanic acid. The main objective of this study was to increase the level of clavulanic acid production by *S. clavuligerus*, using UV irradiation. 

## Experimental

*Microorganism and cultivation*

*Streptomyces clavuligerus *(DSM 738) was revitalized on GYM- broth and maintained on GYM-Agar. Sodium chloride solution (0.9%) containing tween-80 (0.2%) was poured onto GYM-Agar medium, on which *S. clavuligerus *was grown for 7 days at 28 °C. The spores and aerial mycelia were scraped off the agar surface, using a sterile loop. After filtering through cotton wool, the spore suspension was serially diluted to 10^2^ CFU mL^-1^ and one mL of this suspension was poured and spread onto each sterile glass plate containing GYM-Agar and caffeine (0.5 g L^-1^) (28). 

*UV Irradiation*

The plates were irradiated with UV light (200-300 nm, 1.7 W m^-2^) for 20 min, at a distance of 8 cm. After the irradiation, in order to protect against photoreactivation, the plates were wrapped with aluminum foil and incubated for 7 days. Finally, the colonies were sub-cultured (7 days, 28 °C) again to express and stabilize the mutants. 

*Bioassay*

A radically modified bioassay method, based on the method developed by Lee et al (7), was used. Tenμµl of the spore suspension (10^2^ CFU mL^-1^), prepared from the resulted culture, was poured into each of the 3 wells (out of 4 wells) in GYM- agar plates. A spore suspension (10^2 ^CFU mL^-1^) of the wild type micro-organism was poured into the 4^th^ well. After an incubation period of 6 days at 28 °C, the plates were overlaid with nutrient-agar containing penicillin G (7 µg mL^-1^) and *Klebsiela pneumoniae *(10^5^ CFU mL^-1^), and at the end again incubated for 24 h at 37 °C. The inhibition zone diameter was measured and compared with the wild type colony. 

*Clavulanic acid production*

The pH of the seed medium (20 mL) consisting of peptone (1%), malt (1%) and glycerol (2%) was adjusted to 7.0 ± 0.1. The medium was inoculated (5%) and incubated (28 °C, 24 h, 220 rpm) in a 100 mL shaking flask. The production medium (50 mL) containing malt extract (3%), glycerol (0.5%), soy flour (0.5%), FeSO_4_ (0.0001%), and MnSO_4_ (0.0001%) was inoculated (5%) with the seed culture and incubated (28 °C, 220 rpm) in a 250 mL shaking flask. 

*HPLC analysis*

Clavulanic acid was quantified by HPLC, using a reversed-phase column (C-18). The mobile phase contained KH_2_PO_4_ (50 mM, 70%, 0.348 mL min^-1^) and methanol (30%, 0.157 mL min^-1^) and the pH was adjusted to 3.2. Samples were derivatized using imidazole. The derivatives were detected at 311 nm (29). 

*Statistical analysis*

Statistical analyses were performed through the Mann-Whitney non-parametric test, using the statistical package SPSS for Windows version 13.0 software (SPSS Inc, Illinois). 

## Results

During the subculturing of *S. clavuligerus *on GYM agar for sporulation, it was observed that by using deep (≈ 8 mm) agar plates denser colonies appeared with more grey pigments. Pale yellow droplets were secreted 6-7 days after the growth, which disappeared after leaving holes on the surface of *S. clavuligerus *colonies for a period of 24 h. This procedure has not been described before.

It seems that a thicker layer of agar in the plates, resulting in a greater population of the microorganism, could cause a higher and faster production of secondary metabolites, possibly containing antibiotics and clavulanic acid. After a while, these droplets might have been diffused in the plates. 

*Development of a bioassay for clavulanic acid*

**Figure 1:**
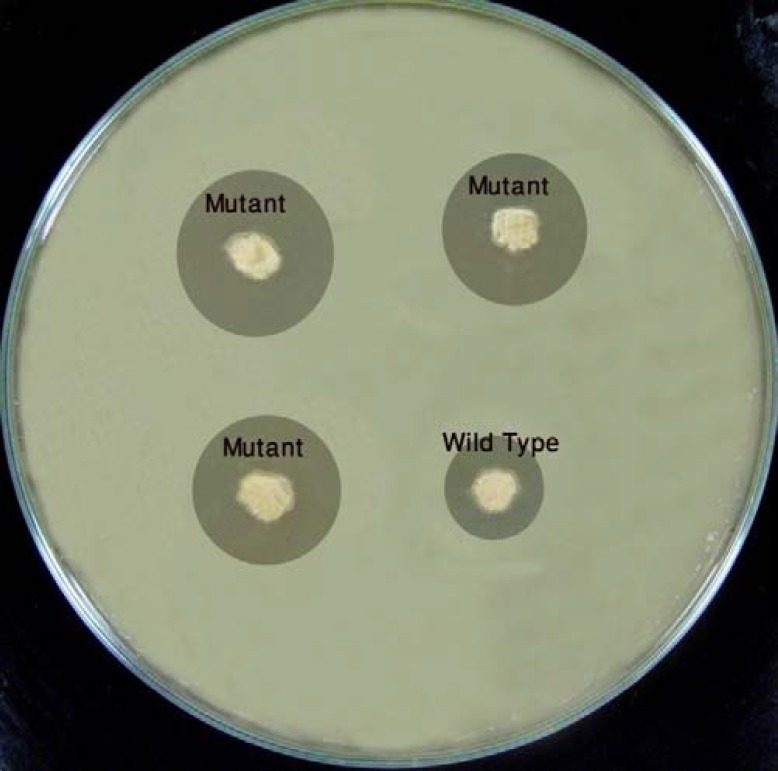
The developed bioassay plates used in this study

In order to screen overproducing mutants, a cheap, quick and fairly reliable bioassay method considering *S. clavuligerus *as clavulanic acid producer, and *K. pneumonia *as the sensitive microorganism was needed. When the bioassay was performed about 4 days after the growth of *S. clavuligerus*, not only the inhibition zone of *K. pneumonia *was not observed, but also a significant overgrowth zone was seen. However, when this assay was performed after 6 days, the inhibition zone was clearly observed (Figure 1).

It might be interpreted that some primary metabolites stimulating the growth are secreted by *S. clavuligerus *colonies during 4 days, which can lead to a faster growth of *K. pneumonia. *On the other hand, after 6 days, secondary metabolites (such as clavulanic acid), inhibiting the growth, might have been produced.

*Screening of overproducing mutants*

**Figure 2:**
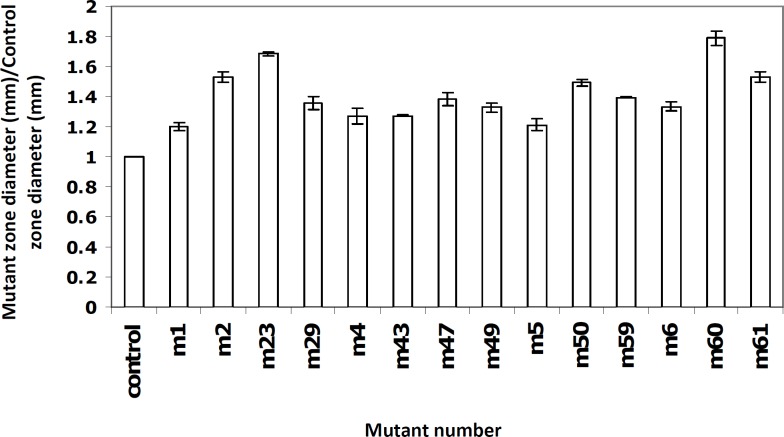
The ratio of mutants inhibition zone diameter over the wild type inhibition zone diameter in selected mutants- Data points have been expressed as mean ± standard error (n = 3).

As mentioned earlier, a bioassay method was used to screen the overproducer mutants. Fourteen (out of 105) mutants showed inhibition zones more than the wild type control, with higher values for the mutants M60, M23 and M61 (Figure 2). Based on the Mann-Whitney statistical test, the differences between groups were found to be significant (P < 0.001).

*Clavulanic acid determination by HPLC*

**Figure 3:**
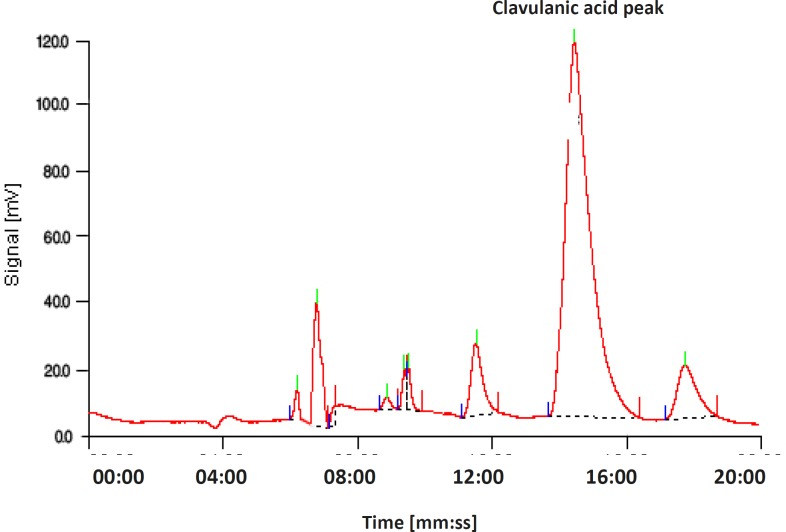
Chromatogram of the products, obtained using a reversed-phase column (C-18). The mobile phase contained KH_2_PO_4_ (50 mM, 70%, 0.348 mL min^-1^) and methanol (30%, 0.157 mL min^-1^) and the pH was adjusted to 3.2. Samples were derivatized using imidazole, and they were detected at 311 nm

**Figure 4:**
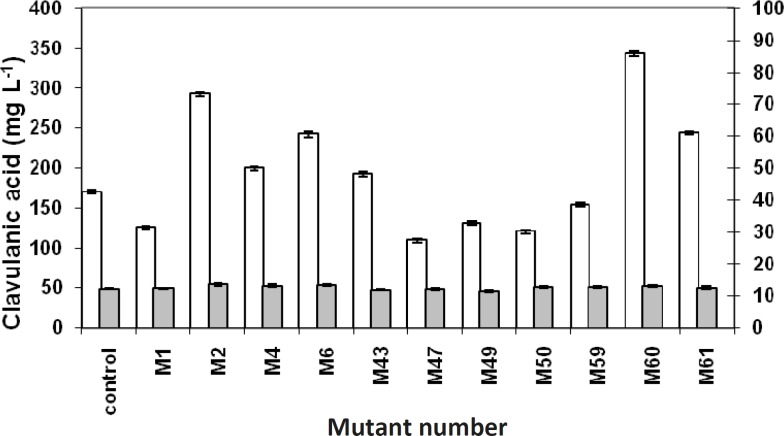
Clavulanic acid versus biomass production. Clavulanic acid concentrations (white columns) were measured by HPLC, and the wet biomass (gray columns) was measured by weighing. Data points have been expressed as mean ± standard error (n = 3).

The bioassay method used did not prove the production of clavulanic acid by the screened mutants. Therefore, we needed to confirm this and determine clavulanic acid concentration. Overproduction of clavulanic acid by the screened mutants was verified by the HPLC measurements (Figures 3 and 4). Intra-day and inter-day variations in precision (CV %) and accuracy (Error %) were found to be acceptable.

**Figure 5:**
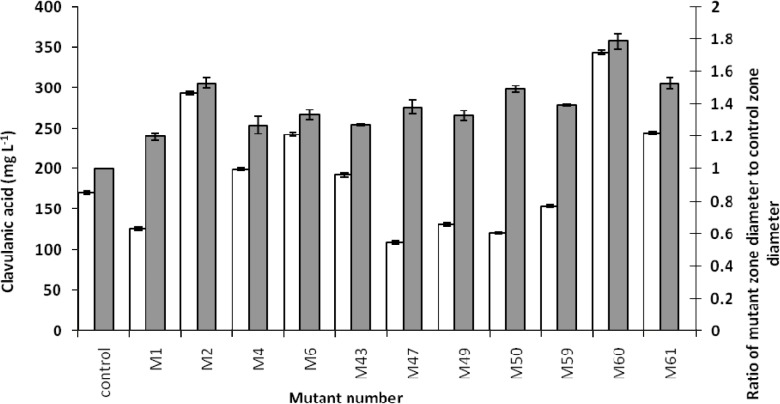
Clavulanic acid production versus the ratio of mutant zone diameter to control zone diameter. Clavulanic acid concentrations (white columns) were measured by HPLC, and the ratio of mutant zone diameter to control zone diameter (gray columns) was measured by Vernier caliper and division. Data points have been expressed as mean ± standard error (n = 3).

Comparing the concentrations of produced clavulanic acid with the biomasses of related mutants, clavulanic acid concentrations were found to be different among the various mutants. However, there was no considerable difference in the biomass concentrations (Figure 4). It might be interpreted that the overproduction of clavulanic acid, following the mutations, is not the result of overgrowth. This could be due to the changes in the metabolic pathway of the microorganism, resulting in the overproduction of clavulanic acid There was an analogy between the bioassay and HPLC method (Fig. 5), except in few cases, which might be due to the overproduction of other growth limiting factors. 

Productivity of the selected mutants was also calculated (Table 1). 

**Table 1:** Productivity of four selected mutants, investigated in this study

**Mutant number**	**Clavulanic acid concentration** **(mg L** ^-1^ **)**	**Production time** **(h)**	**Productivity** **(mg L** ^-1^ **h**^-1^**)**
M2	293.11	72	4.07
M6	242.67	72	3.37
M60	343.87	72	4.77
M61	244.38	72	3.39

## Discussion

The production of clavulanic acid was successfully increased 1.8 times by UV mutagenesis. It seems that the mutations occurred in the genes which were involved in the metabolic pathway of clavulanic acid production, but not in the primary metabolites responsible for growth. These mutants can be used industrially for production of clavulanic acid. A group of researches (7) increased clavulanic acid production up to 8 folds by UV mutagenesis and medium optimization. We increased it up to 1.8 folds, using random mutagenesis, which can be continued more considering the fact that “mutation is an accidental event”. In another study (30), we also inserted *claR *gene in *E. coli *XLI blue, using *PZSclaR *plasmid, as a vector to increase clavulanic acid production. In the near future we will combine these methods, and by using the medium optimization, it is hoped to increase the productivity. 
